# Assessment of levels of awareness towards blood donation in Saudi Arabia

**DOI:** 10.3934/publichealth.2018.3.324

**Published:** 2018-09-04

**Authors:** Saleh Hadi Alharbi, Fahad A. Alateeq, Ibrahim Bin Ahmed, AbdulRahman Ali A. Alsogair, Yousef Duhaim A. Al-Rashdi, Thamer Z. Aldugieman, Hussain Gadelkarim Ahmed

**Affiliations:** 1 Faculty of Medicine, Al Imam Mohammad Ibn Saud Islamic University, Riyadh, Kingdom of Saudi Arabia (KSA); 2 College of Medicine, University of Hail, Saudi Arabia

**Keywords:** blood donation, Saudi Arabia, awareness

## Abstract

**Background:**

blood products is an interesting term as all blood and blood transfusion procedures carry risk even with advance screening of donors and donated blood. In Saudi Arabia voluntary donors are either the only source or not, the other source is paid donors and the lack of volunteers represents a major challenge. This is usually attributed to low community awareness regarding voluntary blood donation. Therefore, the objective of this study was to assess the levels of awareness towards blood donation in northern Saudi Arabia.

**Methodology:**

in this descriptive cross sectional survey, data about blood donation were obtained from 717 Saudi volunteers living in the city of Hail, Saudi Arabia. A Purposeful questionnaire was designed and used for obtaining of the necessary data.

**Results:**

When asking the participants to rate the level of awareness about blood donation in Saudi Society, the majority of participants categorized the levels as good followed by very good and poor, representing 48.8%, 29.3%, and 22% participants, respectively.

**Conclusion:**

the broad concept of blood donation is still poor in Saudi Arabia. Further educational plans are needed to raise the level of awareness and increase the perception of blood donation among Saudi population. Efforts should be made to involve females in blood donation duties.

## 1. Introduction

Blood donation (BD) is considered as a typical altruistic behavior, and recruitment/retention campaigns give emphasis to altruism. Here, a benevolence hypothesis for blood donation (both the donor and recipient benefit) rather than the altruism hypothesis (only the recipient gains) is proposed [1]. While BD is traditionally described as a behavior motivated by pure altruism, the assessment of altruism in the BD literature has not been theoretically informed [2]. The World Health Organization (WHO) encourages that blood donation becomes voluntary and unremunerated [3]. The benevolence hypothesis (both donor and recipient gain) suggests that blood donors, compared to non-blood donors have a general altruistic motivational preference based on warm glow (i.e., “I donate because it makes me feel good”) [4]. The benevolence hypothesis is supported, suggesting that blood donor motivation is partly selfish. Blood donation campaigns should focus on benevolent rather than purely altruistic messages [1].

In a meta-analysis sought to identify the strongest antecedents of blood donation behavior and intentions. It synthesized the results of 24 predictive correlational studies of donation behavior and 37 studies of donation intentions. The antecedents were grouped into six categories: (1) the Theory of Planned Behavior (TPB) and its extensions, (2) prosocial motivation, (3) affective expectations, (4) donor site experience, (5) past donation behavior, and (6) donor demographics [5]. Retention of blood donors has benefits over recruitment of new blood donors. Retention is defined as preventing donors from lapsing and eventually becoming inactive. Studies have found that intention to donate, attitudes towards blood donation and self-efficacy (does one feel capable of donating blood) are predictors of blood donation [6].

In the Kingdom of Saudi Arabia (KSA) the blood transfusion service is basically a hospital-based blood banking system where blood banks are responsible for the whole service, including the recruitment of donors, testing donated blood. The source of donated blood is a combination of direct donation (mainly relatives, friends and workmates of patients), and a growing number of voluntary non-remunerated donors [7]. However, there are other possibilities including blood donation campaigns, regional blood banks and Red Crescent.

Prevalence of blood donation was less than satisfactory among the Saudi public, probably due to misconceptions, poor knowledge, and unfavorable attitude to donation. Educational programs are necessary to increase the level of knowledge and improve the attitude of the Saudi public toward blood donation [8]. Therefore, the objective of the present study was to assess the levels of awareness toward blood donation in northern Saudi Arabia.

## 2. Materials and methods

### 2.1. Ethical consent

Each participant was asked to sign a written ethical consent during the questionnaire's interview. The informed ethical consent form was designed and approved by the ethical committee of the College of Medicine (University of Hail, Saudi Arabia) Research Board.

In this cross sectional survey, data about blood donation were obtained from 717 Saudi volunteers living in the city of Hail, Saudi Arabia. Participants were randomly selected by simple random regardless to age, gender and education or occupation. The participants were recruited from different public gathering places, including educational entities, clubs and others. Data were obtained during face-to-face interview.

A Purposeful questionnaire was designed and used for obtaining of the necessary data. The following information were obtained from each participant: age, sex, education level and occupation sector. Questions regarding awareness about blood donation were also included, which comprised: The source of knowledge about blood donation, rate the level of awareness about blood donation in Saudi Society, does blood donation has beneficial effects for the donor? Donation decreases heart and arterial diseases, during donation you can detect hidden diseases, does blood donation is an important issue, if there is announcement for rare blood group and you possess it will go? The frequency of blood donation, do you know the benefits of blood donation, donation can activate blood circulation and blood renewal, during donation you can detect diseases, does the blood donation important? The factors motivated for blood donation are?

The term “free work” in the study means: self-employed and jobless. Public gathering Include: what is locally called “Diawania”, clubs etc.

### 2.2. Data analysis

Statistical Package for Social Sciences (version 16) was used for analysis and to perform Pearson Chi-square test for statistical significance (*p* value). The 95% confidence level and confidence intervals were used. *p* value less than 0.05 was considered statistically significant.

## 3. Results

This study investigated 717 volunteers, their ages ranging from 15 to 67 years with a mean age of 30 years. Out of the 717 study subjects, 442 (61.6%) were males and 275 (38.4%) were females, giving males' females' ration of 1.61 to 1.00.

**Figure 1.**
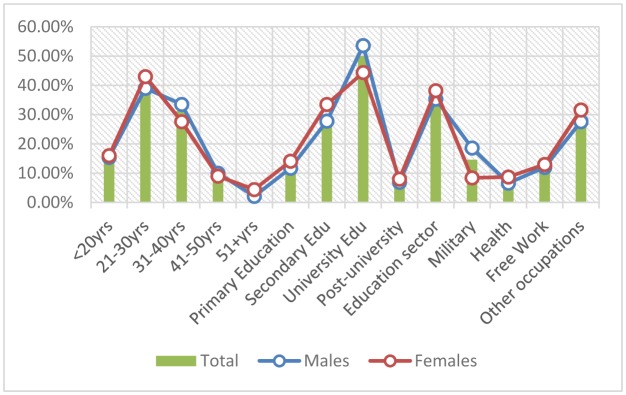
Description of study subjects by sex and demographical characteristics.

As described in Table 1, the great majority of the participants were at age group 21–30 years representing 291/717 (40.6%), followed by age ranges 31–40, <20 and 41–50 constituting 224/717 (31.2%), 112/717 (15.6%), and 69/717 (9.6%), respectively. For males, most of participants were at age group 21–30 years followed by 31–40, <20, and 41–50 years representing 173, 148, 68, and 44, respectively. For females, most of participants were at age range 21–30 years followed by 31–40, <20, and 41–50 constituting 118, 76, 44 and 25, in this order, as shown Figure 1.

With regard to education level, the majority of the participants were at university level followed by secondary, primary and post-university constituting 359/717 (50%), 215/717 (30%), 91/717 (12.7%) and 52/717 (7.3%), respectively. For both males and females the majority of participants were found with university, secondary, primary and post-university levels, decreasingly in this order, as indicated in Table 1, in Figure 1.

**Table 1.** Distribution of the study population by demographical characteristics.

Variable	Category	Males	Females	Total
*Age*				
	<20 years	68	44	112
	21–30	173	118	291
	31–40	148	76	224
	41–50	44	25	69
	51+	9	12	21
	Total	442	275	717
*Education*				
	Primary	52	39	91
	Secondary	123	92	215
	University	237	122	359
	Post-university	30	22	52
	Total	442	275	717
*Occupation*				
	Education	156	105	261
	Military	82	23	105
	Health	29	24	53
	Free work	53	36	89
	Others	122	87	209
	Total	442	275	717

With regard to the occupation, most of participants were at education sector followed by scattered jobs, military, free work and health, representing 261/717 (36.4%), 209/717 (29%), 105/717 (14.6%), 89/717 (12.4%) and 53/717 (7.4%), respectively. For males, most of the study subjects were at education sector followed by others, military, free work and health decreasingly, in this order. For females most of the participants were at education sector followed by others, free work, health and military escalating in this order, as indicated in Table 1, Figure 1.

**Figure 2.**
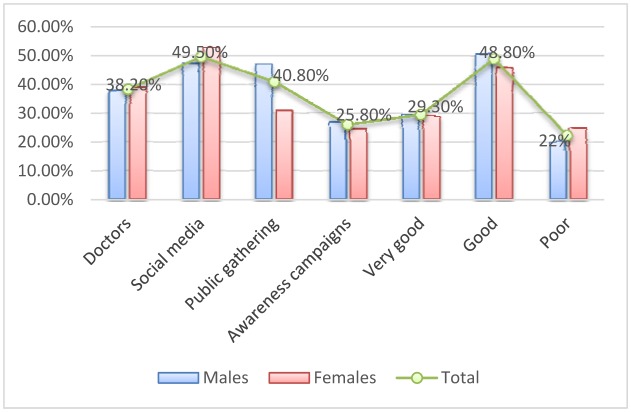
Description of the study population by sex and knowledge about blood donation.

With regard to the source of knowledge about the issue of blood donation, most participants got it from social media followed by public gathering, doctors, and awareness campaigns, representing 355, 293, 274 and 185 participants correspondingly. For males most of them got their knowledge from social media followed by public gathering, doctors and awareness campaigns, constituting 209, 208, 167, and 118 participants, respectively. For females, most of them got their knowledge from social media followed by doctors, public gathering and awareness campaigns, representing 146, 107, 85 and 67 participants, in this order, as indicated in Table 2, Figure 2.

On the other hand when asking the participants to rate the level of awareness about blood donation in Saudi Society, the majority of participants categorized the level as good followed by very good and poor, representing 350, 210, and 157 participants, respectively. Both males and females have rated the levels of awareness, the highest was good followed by very good and poor, as indicated in Table 2, Figure 2.

**Table 2.** Distribution of the study population by sex and knowledge about blood donation.

Variable	Category	Males	Females	Total
*The source of knowledge about blood donation*				
	Doctor	167	107	274
	Social media	209	146	355
	Public gathering	208	85	293
	Awareness campaigns	118	67	185
*Rate the level of awareness about blood donation in Saudi Society*				
	Very good	130	80	210
	Good	224	126	350
	Poor	88	69	157

Table 3 summarized the distribution of the study population by sex and level of awareness about beneficial effects of blood donation. When asking the participants “Is the blood donation has beneficial effects to the donor?” about 406/717 (56.6%) answered “Yes”. Out of the 406 “Yes”, 244/442 (55.2%) were males and 162/275 (59%) were females. When asking the participants “Whether blood donation can activate blood circulation and blood renewal?” around 537/717 (74.9%) answered “Yes”. Out of the 537 “Yes”, 328/442 (74.2%) were males and 209/275 (76%) were females. When asking the participants “During donation you can detect hidden diseases?” about 431/717 (60%) and answered “Yes”. Out of the 431 “Yes”, 258/442 (58.4%) were males and 173/275 (63%) were females. There was no significant values between men and women regarding level of awareness *p* < 0.33.

**Table 3.** Distribution of the study population by sex and level of awareness about beneficial effects of blood donation.

Variable	Category	Males	Females	Total
*Does the blood donation has beneficial effects to the donor?*				
	Yes	244	162	406
	No	198	113	311
*Donation can activate blood circulation and blood renewal*				
	Yes	328	209	537
	No	114	66	180
*Donation decreases heart and arterial diseases*				
	Yes	302	176	478
	No	140	99	239
*During donation you can detect hidden diseases*				
	Yes	258	173	431
	No	184	102	286
	Total	442	275	717

The distribution of the study population by sex and perception about blood donation was summarized in Table 4. When asking the participants “the blood donation important issue?” approximately 648/717 (90.4%) answered “Yes”. Out of the 648 “Yes”, 404/442 (91.4%) were males and 244/275 (88.7%) were females.

**Table 4.** Distribution of the study population by sex and perception about blood donation.

Variable		Category	Males	Females	Total
*Does the blood donation important issue*					
	Yes		404	244	648
	No		38	31	69
*If there is announcement for rare blood group and you possess it will go?*					
	Yes		278	179	457
	May be		89	52	141
	No		74	44	118
*The frequency of blood donation*					
	Yes every 3 months		23	20	43
	Yes every 6 months		13	9	22
	Yes once a year		25	15	40
	No rarely donate		164	65	229
	Total		225	109	334
*The motivated for blood donation is?*					
	Religious duty		249	153	402
	Humanity duty		93	53	146
	National duty		28	10	38
	Mandatory duty		13	14	27
	All		59	45	104
	Total		442	275	717

When asking the participants “If there is announcement for rare blood group and you possess it will go?” about 457/717 (63.7%) and answered “Yes”, 141/717 (19.7%) answered “May be”. Out of the 457 “Yes”, 278/442 (63%) were males and 179/275 (65%) were females. Out of the 141 “May be”, 89/442 (20%) were males and 52/275 (19%) were females.

**Figure 3.**
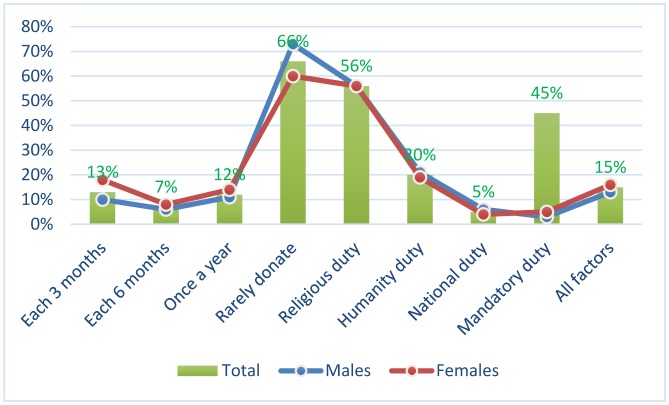
Description of the study population by sex and frequency of blood donation and motivated factors.

With regard to the frequency of blood donation, the majority of participants were rarely donate representing 229/334 (68.6%) followed by those use to donate every 3 months and each 6 months constituting 43/334 (12.9%) and 40/334 (12%), respectively, as indicated in Table 4, Figure 3.

With regard to the motivation for blood donation, the great majority of participants considered blood donation as religious duty constituting 404 participants followed by humanity duty, and those considered all mentioned factors, representing 146 and 104 participants, respectively as shown in Table 4, Figure 3. There was no significant values between men and women regarding perception about blood donation *p* < 0.27.

## 4. Education

The distribution of the study population by education and knowledge about blood donation was summarized in Table 5. Regarding the source of knowledge, most of those with primary level of education got their knowledge from social media followed by public gathering representing 50 and 38 participants, respectively. For secondary level, most participants got their knowledge from social media and doctors. For university level, most participants got their knowledge from social media and public gathering. For Post-university level, most participants got their knowledge from social media and doctors. With regard to the rating the level of awareness about blood donation in Saudi Society; those with primary level of education rated it as very good (n = 35) and poor (n = 33). Secondary level rated it poor (n = 81) and very good (n = 77). University level rated it poor (n = 208) followed by very good (n = 89). Post-university rated it poor (n = 28) followed by good (n = 15).

**Table 5.** Distribution of the study population by education and knowledge about blood donation.

Variable	Category	Primary N = 91	Secondary N = 215	University N = 359	Post-university N = 52	Total
*The source of knowledge about blood donation*						
	Doctor	34	94	122	24	274
	Social media	50	112	169	24	355
	Public gathering	38	79	156	20	293
	Awareness campaigns	19	51	98	17	185
*Rate the level of awareness about blood donation in Saudi Society*						
	Very good	35	77	89	9	210
	Good	23	57	62	15	157
	Poor	33	81	208	28	350

Table 6 summarized the distribution of the study population by education and level of awareness about beneficial effects of blood donation. When asking them “whether regular blood donation has beneficial effects to donor” about 220/359 (61.3%), 119/2015 (55.3%), 27/52 (52%) and 40/91 (44%) of the university, secondary post-university and primary levels, respectively, have answered “Yes”. On asking them “whether regular blood donation can activate blood circulation and blood renewal” around 74.7%, 77.2%, 73.3%, and 77% of the primary, secondary, university and post-university, respectively, have agreed. On asking them “whether regular blood donation decreases heart and arterial diseases” around 50.5%, 62.8%, 72.7%, and 69.2% of the primary, secondary, university and post-university, respectively, have agreed. On asking them “whether regular blood donation can detect hidden diseases” around 60.4%, 56.7%, 61.6%, and 63.5% of the primary, secondary, university and post-university, respectively, have agreed. These findings showed that the level of awareness increase with elevation of education level and this was found to be statistically significant *p* < 0.001.

**Table 6.** Distribution of the study population by education and level of awareness about beneficial effects of blood donation.

Variable	Category	Primary N = 91	Secondary N = 215	University N = 359	Post-university N = 52	Total
*Do you know the benefit of blood donation*						
	Yes	40	119	220	27	406
	No	51	96	139	25	311
	Total	91	215	359	52	717
*Donation can activate blood circulation and blood renewal*						
	Yes	68	166	263	40	537
	No	23	49	96	12	180
*Donation decreases heart and arterial diseases*						
	Yes	46	135	261	36	478
	No	45	80	98	16	239
*During donation you can detect diseases*						
	Yes	55	122	221	33	431
	No	36	93	138	19	286
	Total	91	215	359	52	717

The distribution of the study population by education and perception about blood donation was summarized in Table 7. On asking them “whether blood donation important” around 90%, 90.7%, 90.3%, and 90.4% of the primary, secondary, university and post-university, respectively, have indicated “Yes”.

On asking them “If there is announcement for rare blood group and you possess it will go?” around 58%, 65.6%, 63.8%, and 65.4% of the primary, secondary, university and post-university, respectively, have indicated “Yes”. Moreover, around 26.3%, 22.3%, 16.4%, and 21.2% of the primary, secondary, university and post-university, respectively, have indicated “May be”. With regard to the frequency of blood donation, about 4.4%, 3.7%, 7.8%, and 5.7% of the primary, secondary, university and post-university, respectively, use to donate each 3 months. About 0%, 1.9%, 3.9%, and 7.8% of the primary, secondary, university and post-university, respectively, use to donate each 6 months. About 2.2%, 6.5%, 5.8%, and 11.5% of the primary, secondary, university and post-university, respectively, use to donate once a year. About 30.7%, 26.5%, 34.8%, and 30.7% of the primary, secondary, university and post-university, respectively, were rarely donate.

**Figure 4.**
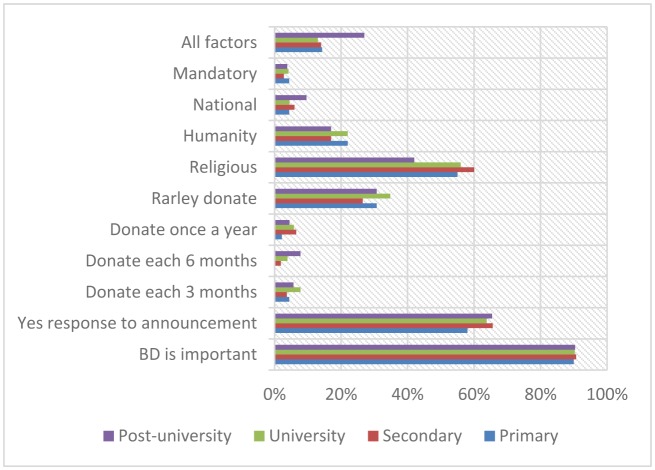
Distribution of the study population by education and perception about blood donation.

On asking them about the motivated factors for blood donation the majority of the participants have indicated religious (56.3%). Out of 404 pointed to religious motivated factor, about 55%, 60%, 56%, and 42% were among the primary, secondary, university and post-university, respectively. Out of 146 pointed to humanity motivated factor, about 22%, 17%, 22%, and 17% were among the primary, secondary, university and post-university, respectively, as indicated in Table 7, Figure 4.

**Table 7.** Distribution of the study population by education and perception about blood donation.

Variable	Category	Primary N = 91	Secondary N = 215	University N = 359	Post-university N = 52	Total
*Is blood donation important*						
	Yes	82	195	324	47	648
	No	9	20	35	5	69
	Total	91	215	359	52	717
*If there is announcement for rare blood group and you possess it will go?*						
	Yes	53	141	229	34	457
	May be	24	48	59	11	141
	No	14	26	71	7	118
*The frequency of blood donation*						
	Yes each 3 months	4	8	28	3	43
	Yes each 6 months	0	4	14	4	22
	Yes once a year	2	14	21	6	43
	No rarely donate	28	57	125	16	226
*The factors motivated for blood donation is?*						
	Religious duty	50	129	201	22	402
	Humanity duty	20	37	80	9	146
	Nationality duty	4	13	16	5	38
	Mandatory duty	4	6	15	2	27
	All	13	30	47	14	104
	Total	91	215	359	52	717

## 5. Occupation

The distribution of the study population by occupational sector and awareness towards blood donation was summarized in Table 8. Regarding the source of knowledge about blood donation; for those in educational sector, most of them gain their knowledge from social media and public gathering representing 139/261 (53.3%) and 114/261 (43.7%) respectively. For those in military sector, most of them gain their knowledge from public gathering and social media representing 69/105 (65.7%) and 41/105 (39%) respectively. For those in health sector, most of them gain their knowledge from social media and doctors representing 28/53 (52.8%) and 21/53 (39.6%) respectively. For those in free work sector, most of them gain their knowledge from social media and doctors representing 43/89 (48.3%) and 36/89 (40.4%) respectively, as indicated in Table 8.

**Table 8.** Distribution of the study population by occupation sector and knowledge about blood donation.

Variable	Category	Education N = 261	Military N = 105	Health N = 53	Free work N = 89	Others N = 209	Total N = 717
*The source of knowledge about blood donation*							
	Doctor	112	31	21	36	74	274
	Social media	139	41	28	43	104	355
	Public gathering	114	69	20	34	56	293
	Awareness campaigns	87	21	19	23	35	185
*Rate the level of awareness about blood donation in Saudi Society*							
	Very good	68	24	12	25	81	210
	Good	61	11	8	24	53	157
	Poor	132	70	33	40	75	350
*Do you know the benefit of blood donation*							
	Yes	140	76	31	43	116	406
	No	121	29	22	46	93	311
	Total	261	105	53	89	209	717
*Donation can activate blood circulation and blood renewal*							
	Yes	213	89	37	65	133	537
	No	48	16	16	24	76	180
*Donation decreases heart and arterial diseases*							
	Yes	198	78	33	57	112	478
	No	63	27	20	32	97	239
*During donation you can detect diseases*							
	Yes	173	73	32	49	104	431
	No	88	32	21	40	105	286
	Total	261	105	53	89	209	717

Regarding rating the level of awareness about blood donation in Saudi Society, for educational sector, military sector, health sector and free work sector, the majority of participants indicated it as poor, representing 132/261 (50.6%), 70/105 (66.7%), 33/53 (62.3%), and 40/89 (45%), correspondingly. However, for others group sector, the majority of participants indicated it as very good followed by poor constituting 81/209 (38.8%) and 75/209 (36%), in this order, as indicated in Table 8. On the other hand, the level of the awareness was found to be influenced by level of education and occupation more than age and sex.

As shown, Table 8 summarized the distribution of the study population by occupation sector and level of awareness about beneficial effects of blood donation. On asking them “Do you know the benefit of blood donation?” about 140/261 (53.6%), 76/105 (72.4%), 31/53 (58.5%), 43/89 (48.3%) and 116/209 (55.5%), of the education, military, health, free work and others scattered jobs, respectively, have expressed “Yes”. On asking them “Do donation can activate blood circulation and blood renewal?” about 213/261 (81.6%), 89/105 (85%), 37/53 (70%), 65/89 (73%) and 133/209 (63.6%), of the education, military, health, free work and others scattered jobs, respectively, have expressed “Yes”. On asking them “Do donation decreases heart and arterial diseases?” about 198/261 (75.7%), 78/105 (74.3%), 33/53 (62.3%), 57/89 (64%) and 112/209 (53.6%), of the education, military, health, free work and others scattered jobs, respectively, have expressed “Yes”. On asking them “Whether During donation you can detect diseases?” about 173/261 (66.3%), 73/105 (70%), 32/53 (60%), 49/89 (55%) and 104/209 (50%), of the education, military, health, free work and others scattered jobs, respectively, have expressed “Yes”.

Table 9 summarized the distribution of the study population by occupation sector and perception about blood donation. On asking them “whether blood donation is important”, only 24, 6, 5, 7, and 27 of the education, military, health, free work and other sectors, in this order, have agreed. On asking them “If there is announcement for rare blood group and you possess it will go?” around 168, 78, 32, 51, and 128 of the education, military, health, free work and other sectors, in this order, have agreed. With regard to the frequency of donation, about 23, 10, 3, 3, and 4 of the education, military, health, free work and other sectors, in this order, use to donate each 3 months. About 137, 25, 25, 50, and 142 of the education, military, health, free work and other sectors, in this order, rarely donate (see Table 9). When asking them about the motivated factors for blood donation; the majority of participants in all occupations have pointed out to religious (see Table 9), and this was found to be statistically significant *p* < 0.0001.

**Table 9.** Distribution of the study population by occupation sector and perception about blood donation.

Variable	Category	Education	Military	Health	Free work	Others	Total
*Is blood donation important*							
	Yes	24	6	5	7	27	69
	No	237	99	48	82	182	648
	Total	261	105	53	89	209	717
*If there is announcement for rare blood group and you possess it will go?*							
	Yes	168	78	32	51	128	457
	May be	42	10	10	25	54	141
	No	51	16	11	13	27	118
*The frequency of blood donation*							
	Yes each 3 months	23	10	3	3	4	43
	Yes each 6 months	11	3	2	4	2	22
	Yes once a year	17	10	3	6	4	40
	Rarely donate	137	25	25	50	142	379
*The factor motivated for blood donation is?*							
	Religious duty	118	67	25	54	138	402
	Humanity duty	50	14	13	17	52	146
	Nationality duty	22	1	2	2	11	38
	Mandatory duty	15	2	5	1	4	27
	All	56	21	8	15	4	104

## 6. Discussion

The requirement for blood transfusion is very high in Saudi Arabia, due to high rates of road traffic injuries [9], beside regular need in health services. Thus, blood donation represents an important issue in the health system in Saudi Arabia. Therefore, the aim of the present study was to assess the level of awareness towards blood donation in a random community based study in Northern Saudi Arabia. This study included individuals with diverse demographical characteristics in term of sex, age, education and occupational sectors.

With regard to the source of knowledge about blood donation, the most effective sources were social media and public gathers rather than the awareness efforts accompanying the blood donation campaigns. Beside the traditional public gathering places such as clubs, education entities and cafés, in Saudi Arabia there are other public gathering entities, such as mosques and Estraha (special building for spending leisure time with friends, particularly at night or during vacations). However, the implementation of effective communication interventions represents a major public health issue. Nevertheless, persuasive media campaigns appear to have little effect on behaviors. Even though non-donors hold a positive attitude towards blood donation, they are not inclined to donate. As an alternative to producing behavioral changes, many recent studies have shown the superiority of binding communication over persuasive communication [10].

In the present study, the majority of the surveyed individuals believed that the level of awareness toward blood donation is good among Saudi people. This might be implicit in term of response, since the majority of Saudi people think that helping others is religious obligation. Religion is deeply rooted in the Saudi society and it was assumed to be a major motivating factor for blood donation [7]. Consequently, other factors, which motivates blood donation should be implemented within awareness campaigns in Saudi Arabia.

In context of the beneficial effects of blood donation, high proportions of the participants have emphasized that blood donation has beneficial effects to the donor. According to some studies blood donors have a lower risk of cardiovascular incidents. This may be associated with the risk of cardiovascular disease reported by some authors, as well as with the oxidative changes caused by iron [11]. Regular blood donation may be protective against cardiovascular disease as reflected by significantly lower mean total cholesterol and low-density lipoprotein levels in regular blood donors than in non-donors [12]. Endothelial dysfunction, secondary to systemic inflammation and oxidative stress, is known to play a major role in the development and progression of atherosclerosis. It was found that regular blood donation is associated with improved endothelial function in healthy adult males [13]. Though there reasonable number of Saudi population know the beneficial effects of blood donation, still there is a need to extend this knowledge to large section of Saudi people.

In the present study we found that the majority of participants were rarely donate blood, particularly females. Saudi females constitute less than 5% of blood donors. The attitude of Saudi female students towards blood donation is positive and few misconceptions that emerged could be corrected by health awareness campaigns [14]. However, similar study from Saudi Araba has concluded that the prevalence of blood donation was less than satisfactory among the Saudi public, probably due to misconceptions, poor knowledge, and unfavorable attitude to donation. Educational programs are necessary to increase the level of knowledge and improve the attitude of the Saudi public toward blood donation. Providing mobile blood collection units nearer to individuals' places of work to reduce their time costs of donating is a necessity [8].

With regard to education, the great majority of participants in the present study were from relatively higher educated people. This may the risk of getting more positive attitude towards blood donation than those selected as cross-sectional varieties. This might be considered as a limitation in the present study.

On the other hand, in the current study we measured different blood donation related factors in association with influence of occupation. Also we found that most participants were clustering within certain occupation, particularly education sector and military. With our strong proposing that some of these occupations have influence in raising blood donation awareness, but our sample selection may not enable us to draw specific guessing in this context.

Although the present study has provided a huge data for future planning of blood donation strategies, as well as stimulating other studies, it has some limitations, particularly in sampling planning.

## 7. Conclusions

The broad concept of blood donation is still poor in Saudi Arabia. Further educational plans are needed to raise the level of awareness and increase the perception of blood donation among Saudi population. The best use of social media and public gathering should be implemented in blood donation strategies. Efforts should be made to involve females in blood donation duties.
